# The Effect of Clay‐ and Yeast‐Based Mycotoxin Binder on Performance, Lymphoid Organs, Blood Parameters, Immune Response and Haematology Parameters of Broilers Exposed to Aflatoxin B_1_

**DOI:** 10.1002/vms3.70625

**Published:** 2025-10-24

**Authors:** Payman Mahmoudi Nasr, Kaveh Jafari Khorshidi

**Affiliations:** ^1^ Department of Animal Science, QaS.C. Islamic Azad University Qaemshahr Iran

**Keywords:** aflatoxin, antibody titre, immunity, liver, toxin binder

## Abstract

**Background:**

Feeding broilers aflatoxin‐contaminated feed can disrupt the bird's performance, immune system and organs such as the liver, kidneys and heart. One possible strategy to minimise the inevitable impact of aflatoxins could be the use of toxic binders in the feed.

**Objectives:**

The present study aimed to investigate the effect of mycotoxin binder magnotox (MAG) in reducing the toxicity of aflatoxin B_1_ (AFB_1_) on performance, lymphoid organs, blood parameters, immune response and haematology parameters of broiler chickens.

**Methods:**

A total of 240 1‐day‐old male broiler chicks of Ross 308 strain with an average 1‐day weight of 43.47 ± 0.28 g were used. This experiment was conducted as a 2 × 2 factorial in a completely randomised design with four treatments and six replications. The experimental diets included two levels of AFB_1_ (0 and 200 µg/kg) and two levels of MAG (0 and 1 g/kg).

**Results:**

The interaction effects of AFB_1_ and MAG showed that adding 1 g/kg MAG to the AFB_1_‐containing diet improved BWG compared to the AFB_1_‐containing diet without MAG at 1–24, 25–42 and 1–42 days of age. Exposure to 200 µg/kg AFB_1_ increased the percentage of liver, kidney and pancreas, and the use of 1 g/kg MAG in the AFB_1_‐containing diet significantly reduced the weight of these organs (*p* < 0.05). Regarding liver enzymes, MAG compensated for the increase in ALP, AST and ALT concentrations caused by AFB_1_ and reduced them similarly to the control treatment (*p* < 0.01). AFB_1_ exposure decreased IgG (24 and 42 days), IgM (24 days) and IgA (42 days) but the use of 1 g/kg MAG in AFB_1_‐contaminated diets was able to compensate for the negative effects of AFB_1_ and significantly increase the concentration of IgG, IgM and IgA. The interaction of MAG and AFB_1_ showed that IBD and ND titres were reduced in AFB_1_‐contaminated diets at 24 and 42 days of age but the use of MAG increased IBD and ND titres (*p* = 0.001).

**Conclusions:**

Overall, broilers fed diets contaminated with 200 µg/kg AFB_1_ showed poor growth performance throughout the period due to impaired immune function and inflammation of immune organs, but supplementation with 1 g/kg MAG counteracted the negative effects caused by AFB_1_ and improved growth performance.

## Graphical abstract

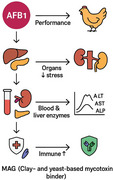

## 1 Introduction

In most parts of the world, poultry chicks are exposed to feeds containing mycotoxins (Abbas et al. 2024). *Aspergillus*, *Fusarium* and *Penicillium* are the three dominant genera in the production of mycotoxins (Hussain 2024). Aflatoxin, a group of furanocoumarins, is considered an important toxic and carcinogenic compound amongst mycotoxins. Aflatoxin B_1_ (AFB_1_) is the most abundant and biologically active type of aflatoxin (Saleemi et al. 2023). Aflatoxins are widely known to be immunotoxic, nephrotoxic and hepatotoxic (Yang et al. 2020), amongst which AFB_1_ is known for its mutagenic and teratogenic effects and high degree of toxicity (Khatoon et al. 2024). For this reason, WHO classified it as a Class I carcinogen in 1993. Prolonged consumption of aflatoxin‐contaminated feed induces inflammatory damage to liver cells and stimulates cancer cells through AF‐DNA adducts that cause liver cancer (Hathout and Aly 2014). Aflatoxins can also disrupt metabolic pathways of a wide range of gut microbiota, thereby causing specific metabolic diseases due to their effects on energy supply (Akinrinmade et al. 2016).

Feeding broiler chickens aflatoxin‐contaminated feed impairs the bird's performance, immune system and organs such as the liver, kidney and heart (Azghadi et al. 2024). At 1 and 2 mg/kg of aflatoxin in the diet of broilers, a reduction in body weight gain (BWG), decreased feed intake (FI) and increased feed conversion ratio (FCR) were observed (Tavangar et al. 2021). Aflatoxin poisoning is associated with changes in blood biochemical parameters, blood parameters, pathological outcomes and immune system activity (Mesgar et al. 2022). Compared to other organs, the gastrointestinal tract is the first site to come into contact with mycotoxins, which makes it more vulnerable to AFB_1_ (Galarza‐Seeber et al. 2016). AFB_1_ alters intestinal morphology (Poloni et al. 2020), which can exacerbate intestinal inflammation and impair the utilisation of essential nutrients (Grenier and Applegate 2013).

The increasing knowledge and awareness of aflatoxin as a potent source of health risks for both humans and farm animals has prompted producers, researchers and government agencies to intensify efforts for preventive management and disinfection technologies to minimise aflatoxin content in food and feed (Galvano et al. 2005). In order to reduce the toxic and economic effects of aflatoxin, regulations and legal limits for aflatoxin in poultry feed have been established. Many countries follow a maximum acceptable level of 20 ppb for aflatoxin in poultry feed (Abidin et al. 2017). Pre‐ and post‐harvest contamination can be reduced by using appropriate agricultural practices. However, contamination is often unavoidable and remains a serious problem associated with many agricultural commodities, emphasising the need for a suitable process to inactivate the toxins (Galvano et al. 2005). Since the early 1990s, studies based on adsorbents have been reported to be effective in minimising aflatoxin contamination in feed (Ibrahim et al. 2000). Removal of aflatoxins from contaminated feeds is an important aspect of nutritional research. Various physical, chemical and biological methods have been used to remove aflatoxins (Rashidi et al. 2020). A possible strategy to minimise the unavoidable effect of aflatoxins could be the use of toxic binders in feed. So far, natural zeolite, bentonite, hydrated sodium calcium aluminosilicate, yeast cell wall, activated carbon, probiotics and plant extracts have been used to reduce the toxicity of aflatoxins in poultry feed (Putra et al. 2024). The asymmetric aluminium ion terminal portion of the aluminosilicate structure can reduce the availability and inhibit its toxicity in chickens by binding to the carbonyl group of AFB_1_ (Elliott et al. 2020). Adding mineral adsorbents to poultry diets significantly reduced aflatoxin toxicity and improved FI and body weight of chickens (Mendoza et al. 2022).

The mycotoxin binder used in this study (Magnotox, Vivan, Mashhad, Iran) is a combination of yeast cell wall, aluminium silicates, mineral adsorbents, mycotoxin‐degrading microorganisms and organic acids. However, whether the commercial mycotoxin‐binder magnotox (MAG) can effectively reduce the toxic effects of AFB_1_ when exposed to broilers remains unknown. The aim of this study was to investigate the effect of MAG in reducing the toxicity of AFB_1_ to broilers.

## 2 Materials and Methods

### 2.1 Birds, Diets and Management

In this research, 240 one‐day‐old male broilers of Ross 308 strain with an average one‐day weight of 43.47 ± 0.28 g were used. This design was implemented as a factorial 2 × 2 in the form of a completely random design with 4 treatments, 6 replications and 10 chickens per replication. Experimental treatments include (1) basal diet, (2) basal diet with 1 g /kg MAG (Magnotox, Vivan, Mashhad, Iran), (3) basal diet with 200 µg/kg AFB_1_ and (4) basal diet with 1 g /kg MAG + 200 µg/kg AFB_1_ (Table 1). The adjusted diets used in the starter (1–10 days), growth (11–24 days) and finisher (25–42 days) periods are presented in Table 2. During the entire breeding period, feed and water were freely provided to the birds. Broiler chickens were housed in wire cages and maintained under 23L:1D for this experiment after receiving continuous light for the first 24 h. The room temperature was maintained at 32°C–34°C during the first 5 days and then gradually decreased by 2°C/wk to reach a final room temperature of 22°C–24°C.

**TABLE 1:** Experimental groups and their respective dietary treatments.

Treatments	Diet description	AFB_1_ (µg/kg)	MAG (g/kg)
1	Basal diet	0	0
2	Basal diet + MAG	0	1
3	Basal diet + AFB_1_	200	0
4	Basal diet + AFB_1_ + MAG	200	1

Abbreviations: AFB_1_, aflatoxin B_1_; MAG, mycotoxin binder magnotox.

**TABLE 2:** Components and chemical compositions of the diets during starter, growth and finisher periods.

Diet ingredients (%)	Starter (Days 1–10)	Grower (Days 11–24)	Finisher (Days 25–42)
Corn	55.97	59.97	66.35
Soybean meal 44%	37.58	34.16	28.43
Soybean oil	1.60	1.57	1.53
Dicalcium phosphate	2.47	2.09	1.65
Calcium carbonate	0.68	0.57	0.46
L‐Lys HCl	0.31	0.29	0.27
DL‐Met	0.35	0.31	0.28
L‐Thr	0.14	0.13	0.11
Sodium bicarbonate	0.10	0.12	0.15
Vitamin and mineral‐premix^a^	0.50	0.50	0.50
Salt	0.30	0.29	0.27
Sum	100	100	100
Calculated composition			
AMEn (Kcal/Kg)	2796	2843	2914
Crude protein (%)	21.62	20.37	18.33
Ether extract (%)	4.03	4.08	4.15
Crude fibre (%)	3.29	3.21	3.10
Linoleic acid (%)	2.20	2.27	2.35
Calcium (%)	0.89	0.77	0.61
Available phosphorus (%)	0.47	0.40	0.33
Sodium (%)	0.17	0.17	0.17
Digestible methionine (%)	0.63	0.58	0.53
Digestible methionine + cysteine (%)	0.94	0.88	0.80
Digestible lysine (%)	1.24	1.14	1.01
Digestible threonine (%)	0.82	0.75	0.67
Digestible arginine (%)	1.23	1.15	1.01
Digestible valine (%)	0.86	0.80	0.72
Digestible isoleucine (%)	0.82	0.75	0.67
DCAB (mEq/kg)	231.76	217.69	199.31

Abbreviations: AMEn, apparent metabolisable energy corrected for nitrogen; DCAB, dietary cation–anion balance.

^a^Provides the following per kg of diet: 4.13 mg retinol, 60.00 µg chole‐calciferol, 30.00 mg Dl‐α‐tocopherol, 3 mg menadione, 2.20 mg thiamine, 8.00 mg riboflavin, 5.00 mg pyridoxine, 11.00 µg cyanocobalamin, 1.50 mg folic acid, 150.00 µg biotin, 25.00 mg calcium pantotenate, 65.00 mg nicotinic acid. Provides the following per kg of diet: 60.00 mg Mn (manganese sulphate), 40.00 mg Zn (zinc oxide), 0.33 mg I (potassium iodate), 80.00 mg Fe (ferrous sulphate), 8.00 mg Cu (copper sulphate), 0.15 mg Se (sodium selenite), 150.00 mg ethoxyquin.

Aflatoxin was produced from rice fermentation by *Aspergillus flavus* NRRL 3357 certified by The Islamic Azad University Laboratory, Qaemshahr Branch, Iran, as described by Shotwell et al. (1966). Briefly, *Aspergillus flavus* NRRL 3357 was cultured on potato dextrose agar (PDA) for 7 days at 28°C. To prepare fungal spore suspension, 0.5 mL of 1% Triton X‐100 was added to the PDA plates using filtered syringes. The spores were then inoculated onto rice and incubated in the dark at 25°C for 12 days to produce aflatoxin (Shotwell et al. 1966).

### 2.2 Performance

FI was provided to chickens daily after weighing. To calculate the amount of FI of each repetition, the amount of feed remaining at the end of each breeding period was deducted from the total feed given during the period. To calculate the BWG of each repetition in each period, the difference between the final weight and the beginning of the breeding period was determined. On Days 1, 10, 24 and 42, all chickens of each experimental unit were weighed as a group (Khalifa et al. 2023). The FCR was calculated in different periods (starter, growth and finisher periods). FCR was calculated by dividing the average FI by the average BWG of chickens for each period. During the experiment, daily and before the allocation of feed, the mortality of each experimental pen was recorded and its weight was recorded. The daily mortality rate was used to determine the chicken day of each experimental unit (Awad et al. 2018).

### 2.3 Relative Weight of Organs

At Day 42 of age, two birds in each replicate were slaughtered after approximately 4 h of feed withdrawal. Before slaughter, the birds selected for dissection were weighed again on a digital scale. Viscera were removed immediately, and the weights of heart, liver, pancreas, bursa of Fabricius, thymus, spleen and kidney were measured using a digital scale after that (0.01 g; KEB 602, China). All of the data regarding internal organ weights were expressed as a percentage of live BW.

### 2.4 Blood Parameters

The same two birds' blood samples (5 mL) were taken from the wing vein on each repetition when they were 42 days old. Serum was extracted from blood samples by centrifuging them for 10 min at 4°C and 3000 × g. Samples of blood were sent to the lab, where they were kept cold until further examination. An autoanalyzer (Abbott Laboratories, Illinois, USA) was used to measure blood parameters such as glucose, triglyceride, cholesterol, total protein, albumin and the activity of aspartate aminotransferase (AST), alanine aminotransferase (ALT) and alkaline phosphatase (ALP) enzymes in blood serum using commercial enzyme kits (Pars Azmoun kits, Pars Azmoun Company, Tehran, Iran) (Benzie and Strain 1996).

### 2.5 Immune Competence

At Days 24 and 42, 12 birds in each treatment (two birds from each cage) were selected to collect blood. An approximately 5 mL blood sample was collected from the wing vein into a non‐heparinised tube, placed at room temperature for 30 min, centrifuged at 3000 g for 10 min and the serum was separated and stored at −20°C until further analysis. According to the Elisa kit instruction, the levels of serum immunoglobulin (Ig) G, IgA and IgM were determined.

All the broiler chickens were vaccinated at 7 days of age intramuscularly through the breast muscle with killed Newcastle disease (ND) and infectious bursal disease (IBD) vaccines which were provided. The ND and IBD live vaccine were used in the drinking water for vaccination at 14 days of age. Twelve birds in each treatment at Days 24 and 42 were selected to collect blood samples (5 mL). The serum collected on Days 24 and 42 was subjected to antibody titre assays against the ND virus and IBD virus. The antibody titre against the ND virus was carried out by haemagglutination followed by a haemagglutination inhibition test. The micro‐test method as described by Allan and Gouch (1974) was used for the detection of HI titres from the serum samples. The antibody titre against IBD was measured using indirect ELISA kit and performed as per the methodology recommended by the manufacturer (Zabiulla et al. 2021).

### 2.6 Haematology Parameters

At 42 days of age, blood samples were collected from the wing vein of two birds per replicate to assess haematology parameters. Blood samples were transferred to venoject tubes containing 0.5 mL of ethylenediaminetetraacetic acid (EDTA) anticoagulant and sent to the laboratory. Haematology indices including white blood cell (WBC) count, red blood cell (RBC) count, haemoglobin, lymphocytes, heterophils, monocytes and eosinophils were determined in the laboratory. A haemocytometer slide was used to count the number of RBCs. Haemoglobin was measured using a Pars Peyvand Company kit (Drabkin) by colorimetric and cyanmethemoglobin methods. After preparing an appropriate blood spread, differential leukocyte count was performed by Giemsa method (Aljohani and Zaman 2024).

### 2.7 Statistical Analysis

Data analysis was done by mixed procedure SAS 9.4 statistical software (SAS 2009) as a 2 × 2 factorial arrangement (two levels of AFB_1_ challenge and two levels of MAG treatment). When interactive effects differed significantly, Duncan's multiple comparisons test was used to separate means. Differences were considered significant at *p* < 0.05.

## 3 Results

### 3.1 Broiler Performance

Table 3 shows the effect of the MAG in AFB_1_‐containing diets on FI, BWG and FCR of birds during the different experimental periods. AFB_1_ exposure significantly reduced FI at 1–24, 25–42 and 1–42 days of age. Broilers fed diets containing 1 g /kg of MAG increased FI over the entire period (1–42 days of age) (*p* = 0.03). The interaction effects of AFB_1_ and MAG showed that the addition of 1 g /kg of MAG to the AFB_1_‐containing diet improved BWG compared to the AFB_1_‐containing diet without MAG at 1–24, 25–42 and 1–42 days of age. There was no interaction effect between AFB_1_ exposure and MAG supplementation on FCR (*p* > 0.05).

**TABLE 3:** Effects of dietary AFB_1_ and mycotoxins binder on growth parameters of broiler chickens.

Items		FI (g)			BWG (g)			FCR		
		1–24	25–42	1–42	1–24	25–42	1–42	1–24	25–42	1–42
Control		59.01	160.85	102.66	40.43a	80.48^a^	57.59^a^	1.46	2.00	1.78
MAG		59.16	162.51	103.45	40.03^a^	83.38^a^	58.61^a^	1.47	1.95	1.76
AFB_1_		52.36	148.47	93.55	35.26^b^	72.22^b^	51.10^b^	1.48	2.05	1.82
MAG+AFB_1_		56.89	160.14	101.14	39.84^a^	82.55^a^	58.14^a^	1.42	1.95	1.74
SEM		1.41	3.86	2.02	0.53	2.11	1.02	0.04	0.06	0.04
The main effect										
AFB_1_	−	59.08	161.68	103.05	40.23	81.93	58.10	1.47	1.97	1.77
	+	54.62	154.30	97.34	37.55	77.38	54.62	1.45	2.00	1.78
MAG	−	55.68	154.66	98.10	37.85	76.35	54.35	1.47	2.02	1.80
	+	58.02	161.32	102.29	39.93	82.97	58.38	1.45	1.95	1.75
*p* value										
AFB_1_		0.006	0.02	0.01	0.001	0.04	0.003	0.73	0.64	0.77
MAG		0.11	0.10	0.03	0.003	0.006	0.001	0.64	0.25	0.26
AFB_1_ × MAG		0.14	0.21	0.11	0.03	0.02	0.009	0.37	0.70	0.44

*Note*: Means within a variable with no common superscript (a, b) differ significantly (*p *< 0.05).

Abbreviations: AFB_1_, 200 µg/kg aflatoxin B_1_; BWG, body weight gain; FCR, feed conversion ratio; FI, feed intake; MAG, 1 g/kg magnotox; SEM, standard error of means.

### 3.2 Organ Characteristics

Table 4 shows the effect of toxin binder in AFB_1_‐containing diets on organ characteristics. The results showed that the percentage of liver, kidney and pancreas was affected by the interaction of AFB_1_ and MAG, and exposure to 200 µg/kg AFB_1_ increased the percentage of liver, kidney and pancreas, while the use of 1 g/kg MAG in the AFB_1_‐containing diet significantly reduced the weight of these organs (*p* < 0.05). Also, chicks fed the AFB_1_‐containing diet had a higher relative heart weight than the control treatment (*p* = 0.03). The relative weight of bursa, thymus and spleen was not affected by the experimental treatments (*p* > 0.05).

**TABLE 4:** Effects of dietary AFB_1_ and mycotoxins binder on relative organs weight (% of BW) of broiler chickens at Day 42.

Treatments		Liver	Heart	Kidney	Pancreas	Bursa	Thymus	Spleen
Control		2.30b	0.601	0.459^ab^	0.224^b^	0.162	0.157	0.114
MAG		2.41^b^	0.594	0.425^b^	0.238^b^	0.130	0.168	0.123
AFB_1_		2.88^a^	0.685	0.566^a^	0.396^a^	0.192	0.174	0.143
MAG + AFB_1_		2.49^b^	0.639	0.411^b^	0.253^b^	0.135	0.176	0.128
SEM		0.12	0.02	0.03	0.03	0.020	0.017	0.013
The main effect								
AFB_1_	−	2.35	0.597	0.442	0.231	0.146	0.163	0.119
	+	2.69	0.662	0.488	0.325	0.164	0.175	0.136
MAG	−	2.59	0.643	0.512	0.310	0.177	0.167	0.128
	+	2.45	0.617	0.418	0.246	0.132	0.171	0.125
*p* value								
AFB_1_		0.01	0.03	0.16	0.01	0.18	0.48	0.07
MAG		0.26	0.36	0.009	0.08	0.09	0.80	0.10
AFB_1_ × MAG		0.05	0.49	0.04	0.03	0.11	0.68	0.57

*Note*: Means within a variable with no common superscript (a, b) differ significantly (*p *< 0.05).

Abbreviations: AFB_1_, 200 µg/kg aflatoxin B_1_; MAG, 1 g/kg magnotox; SEM, standard error of means.

### 3.3 Blood Parameters

The effect of MAG in diets containing AFB_1_ on blood parameters and liver enzymes is reported in Table 5. The interaction effect of MAG and AFB_1_ showed that feeding broilers with diets containing AFB_1_ reduced TP and Alb concentrations, but the use of MAG in diets containing AFB_1_ increased the concentrations of these two parameters (*p* < 0.001). Regarding antioxidant enzymes, MAG also compensated for the increase in ALP, AST and ALT concentrations caused by AFB_1_ and decreased similarly to the control treatment (*p* < 0.01).

**TABLE 5:** Effects of dietary AFB_1_ and mycotoxins binder on serum biochemical parameters and liver enzymes activities of broiler chickens at Day 42.

Treatments		Glu (mg/dL)	Trig (mg/dL)	Chol (mg/dL)	UA (mg/dL)	TP (g/dL)	Alb (g/dL)	ALP (U/L)	AST (U/L)	ALT (U/L)
Control		327.61	113.94	150.84	7.82	6.88^a^	2.34^ab^	1606.63^b^	206.67^b^	3.02^b^
MAG		314.44	127.76	155.63	8.42	6.58^a^	3.28^a^	1666.58^b^	213.25^b^	3.78^b^
AFB_1_		317.20	119.62	148.09	10.92	4.02^b^	2.18^b^	1920.31^a^	302.19^a^	6.28^a^
MAG + AFB_1_		310.87	106.45	149.86	8.80	5.55^a^	2.68^ab^	1648.49^b^	208.55^b^	3.47^b^
SEM		17.81	16.45	10.37	0.85	0.42	0.18	25.09	16.46	0.76
The main effect										
AFB_1_	−	321.06	120.85	153.20	8.12	6.73	2.81	1636.60	209.93	3.40
	+	314.18	113.06	148.94	9.86	4.78	2.43	1786.30	255.37	4.87
MAG	−	322.45	116.77	149.42	9.37	5.44	2.26	1763.34	254.37	4.65
	+	312.68	117.18	152.73	8.61	6.07	2.98	1657.62	210.95	3.62
*p* value										
AFB_1_		0.69	0.63	0.68	0.89	0.01	0.44	0.001	0.01	0.07
MAG		0.58	0.98	0.75	0.15	0.39	0.15	0.007	0.01	0.19
AFB_1 _× MAG		0.85	0.42	0.88	0.08	0.001	0.04	0.001	0.007	0.01

*Note*: Means within a variable with no common superscript (a, b) differ significantly (*p *< 0.05).

Abbreviations: AFB_1_, 200 µg/kg aflatoxin B_1_; Alb, albumin; ALP, alkaline phosphatase; ALT, alanine aminotransferase; AST, aspartate aminotransferase; Chol, cholesterol; Glu, glucose; MAG, 1 g/kg magnotox; SEM, standard error of means; TP, total protein; Trig, triglyceride; UA, uric acid.

### 3.4 Immune Competence

AFB_1_ exposure decreased IgG (24 and 42 days), IgM (24 days) and IgA (42 days), but the use of 1 g of MAG in AFB_1_‐contaminated diets was able to compensate for the negative effects of AFB_1_ and significantly increase the concentration of IgG, IgM and IgA (Table 6). The interaction of MAG and AFB_1_ showed that IBD and ND titres were reduced in AFB_1_‐contaminated diets at 24 and 42 days of age, but the use of MAG increased IBD and ND titres (*p* = 0.001).

**TABLE 6:** Effects of dietary AFB_1_ and mycotoxins binder on serum immunoglobulins, serum antibody response to Newcastle disease and infectious bursal disease of broiler chickens at Days 24 and 42.

		IgG (g/L)		IgM (g/L)		IgA (g/L)		IBD titres (Log_10_)		ND titres (Log_10_)	
Items		24 days	42 days	24 days	42 days	24 days	42 days	24 days	42 days	24 days	42 days
Control		3.84^a^	4.12^ab^	1.56^a^	1.48	2.36	2.28^a^	3.74^a^	3.63^a^	3.47^a^	3.21^a^
MAG		3.81^a^	4.19^a^	1.53^a^	1.50	2.32	2.31^a^	3.69^a^	3.65^a^	3.38^ab^	3.28^a^
AFB_1_		3.66^b^	4.01^b^	1.41^b^	1.42	2.31	2.19^b^	3.43^b^	3.56^b^	3.08^b^	3.02^b^
MAG + AFB_1_		3.83^a^	4.21^a^	1.49^a^	1.45	2.34	2.32^a^	3.72^a^	3.68^a^	3.51^a^	3.19^a^
SEM		0.01	0.02	0.01	0.02	0.02	0.01	0.02	0.01	0.02	0.03
The main effect											
AFB_1_	−	3.82	4.15	1.54	1.49	2.34	2.29	3.71	3.64	3.42	3.24
	+	3.74	4.11	1.45	1.43	2.32	2.25	3.57	3.62	3.29	3.11
MAG	−	3.75	4.06	1.49	1.45	2.33	2.23	3.59	3.59	3.27	3.11
	+	3.82	4.20	1.51	1.47	2.33	2.31	3.70	3.66	3.44	3.24
*p* value											
AFB_1_		0.001	0.06	0.001	0.001	0.21	0.01	0.001	0.17	0.001	0.001
MAG		0.002	0.001	0.16	0.12	0.75	0.001	0.001	0.001	0.001	0.001
AFB_1 _× MAG		0.001	0.03	0.007	0.69	0.22	0.002	0.001	0.02	0.001	0.02

*Note*: Means within a variable with no common superscript (a, b) differ significantly (*p *< 0.05).

Abbreviations: AFB_1_, 200 µg/kg aflatoxin B_1_; IBD, infectious bursal disease; IgA, immunoglobulin A; IgG, immunoglobulin G; IgM, immunoglobulin M; MAG, 1 g/kg magnotox; ND, Newcastle disease; SEM, standard error of means.

### 3.5 Haematology Parameters

Exposure to AFB_1_ increased the number of RBCs, but the use of 1 g/kg of MAG in AFB_1_‐contaminated diets was able to compensate for the negative effects of AFB_1_ and significantly reduce the number of RBCs (Table 7, *p* = 0.006). The interaction of MAG and AFB_1_ showed that the percentage of monocytes increased in AFB_1_‐contaminated diets, but the use of MAG increased the percentage of monocytes (*p* = 0.003).

**TABLE 7:** Effects of dietary AFB_1_ and mycotoxins binder on haematology parameters of broiler chickens at Day 42.

Treatments		WBC	RBC	Haemoglobin	Lymphocytes	Heterophils	Monocytes	Eosinophils
Control		18.79	3.13^b^	8.68	61.08	31.77	1.76^b^	3.71
MAG		18.42	3.01^b^	8.78	60.54	32.77	2.12^ab^	3.07
AFB_1_		18.97	4.56^a^	8.14	60.40	31.00	3.11^a^	3.67
MAG + AFB_1_		18.94	3.32^b^	8.35	60.84	32.05	1.94^b^	3.68
SEM		0.45	0.38	0.50	0.57	0.62	0.12	0.05
The main effect								
AFB_1_	−	18.60	3.07	8.73	60.81	32.27	1.94	3.71
	+	18.95	3.94	8.24	60.62	31.53	2.52	3.67
MAG	−	18.88	3.85	8.41	60.74	31.39	2.43	3.69
	+	18.66	3.17	8.57	60.69	32.41	2.03	3.67
*p* value								
AFB_1_		0.44	0.03	0.34	0.74	0.25	0.01	0.55
MAG		0.66	0.09	0.75	0.93	0.12	0.08	0.98
AFB_1 _× MAG		0.71	0.006	0.91	0.41	0.96	0.003	0.86

*Note*: Means within a variable with no common superscript (a, b) differ significantly (*p *< 0.05).

Abbreviations: AFB_1_, 200 µg/kg aflatoxin B_1_; MAG, 1 g/kg magnotox; RBC, red blood cell; SEM, standard error of means; WBC, white blood cell.

## 4 Discussion

Aflatoxins are the most important mycotoxins of concern to poultry farmers and are considered highly toxic due to their rapid absorption in the intestine (Whitlow et al. 2002). Metabolism of these compounds in the liver produces toxic metabolites that cause liver damage and inhibition of protein synthesis, leading to anorexia (Yunus et al. 2011). The results showed that the concentration of AFB_1_ used in this study reduced FI in broilers. This result is consistent with the results of Nazarizadeh and Pourreza (2019), who reported a reduction in FI in broilers fed a diet containing 2 mg/kg AFB_1_. In a similar study, Lai et al. (2022) showed that 200 µg/kg AFB_1_ in the diet significantly reduced BWG and FI of Arbor Acres broilers and increased FCR from 1 to 21 days of age. Most studies have been conducted in the early rearing stage, as birds present an intense growth process at this stage and its harmful effects are more pronounced (Ma et al. 2015).

The addition of toxin binder to aflatoxin‐free diets did not cause any adverse effects on growth performance compared to the control group, indicating that even with some loss of nutrients due to binding to the adsorbent, as described by Qu et al. (2017), it did not alter performance characteristics. The results of the present study showed that MAG reduced the adverse effects on BWG caused by AFB_1_ in broilers. A previous study reported that the addition of toxin binder (1 g/kg feed) to a diet containing AFB_1_ (0.3 µg/kg feed) reduced the growth inhibitory effects in broilers for 21 days (Wade et al. 2018). The adverse effects of AFB_1_ on BWG are due to anorexia, lethargy, inhibition of protein synthesis and lipogenesis (Rotimi et al. 2017). On the other hand, the reduction in growth due to the presence of aflatoxin in the diet can be associated with a decrease in FI, which was significantly observed in the present experiment and is consistent with previous reports on the negative effect of aflatoxin on performance (Khanian et al. 2019). The decrease in FI and growth rate is due to the reduction in the activity of important enzymes in the digestion of carbohydrates, lipids, proteins and nucleic acids and the impairment and defect in the absorption of some nutrients, and as a result, it is associated with the occurrence of deficiencies of these substances. The improvement in BWG in the toxin binder experimental group is probably due to the interaction between the carbonyl group of aflatoxin and the asymmetric aluminium ion terminal part present in the structure of aluminosilicate compounds, which reduces the availability of the toxin and inhibits its toxicity in chickens (Řehulka et al. 2005).

The improvement in FI and BWG observed in broilers supplemented with MAG can be attributed to the synergistic actions of its multiple active components. Yeast cell wall components, particularly β‐glucans and mannan oligosaccharides, are known to adsorb aflatoxins through hydrogen bonding and van der Waals interactions, thereby reducing their intestinal absorption and systemic circulation (Yiannikouris and Jouany 2002). Aluminium silicates and other mineral adsorbents further contribute by physically binding aflatoxin molecules in the gastrointestinal tract, preventing their transfer into the bloodstream (Phillips et al. 2008). In addition, the inclusion of mycotoxin‐degrading microorganisms can enzymatically biotransform AFB_1_ into less toxic metabolites, lowering its detrimental impact on nutrient utilisation and metabolism (Garcia et al. 2018). Organic acids, on the other hand, may stabilise gut microbiota and improve intestinal morphology, leading to enhanced nutrient absorption. The combined effects of these mechanisms help to preserve normal FI and growth, counteracting the growth‐depressing impact of AFB_1_ exposure.

Changes in the relative weight of immune organs are one of the major changes associated with aflatoxicosis. The increase in relative weight of liver and kidney compared to the control group in this study is consistent with previous research (Neeff et al. 2013). The increase in relative liver weight in AFB_1_‐treated birds can be attributed to AFB_1_‐induced lipid metabolism disorder in the liver with increased lipid content of hepatocytes (Denli et al. 2009). Inhibition of phospholipid and cholesterol synthesis can lead to hepatic lipidosis, which in turn affects lipid transport from the liver (Manegar et al. 2010). In addition, AFB_1_‐induced degenerative, inflammatory and vascular changes in visceral organs may be responsible for the increase in liver weight, and these hypotheses are confirmed in the study of Zabiulla et al. (2021) by histopathological lesions in the liver with severe fatty and lipidic changes. The higher kidney weight in AFB_1_‐treated chickens observed in this study could be due to the development of vacuolar degeneration and renal damage during aflatoxicosis (Sakhare et al. 2007). The increased relative heart weight could be related to previous findings of cardiac enlargement in AFB_1_‐fed broilers (Sharghi and Manafi 2011). The increased relative pancreas weight could be due to damage to the pancreas, as histopathological observations were characterised by severe vascular congestion and mononuclear cell infiltration, which is supported by the findings of Valchev et al. (2014). The significant reduction in relative liver weight and slight reduction in relative heart and pancreas weight with the addition of toxin binder to the AFB_1_‐contaminated diet indicate the potential protection of these organs by toxin binder. The reduction in liver, kidney and pancreas enlargement by MAG supplementation suggests that its active components exerted a protective effect against aflatoxin‐induced organ hypertrophy. AFB_1_ is known to induce hepatomegaly and nephropathy by forming reactive oxygen species and AFB_1_–DNA adducts, which lead to hepatocellular injury and impaired renal function (Wogan 1992; Hathout and Aly 2014). The yeast cell wall fraction in MAG may reduce aflatoxin absorption at the intestinal level, thereby lowering the toxin load reaching the liver and kidneys. Simultaneously, mineral adsorbents such as aluminium silicates can physically bind AFB_1_ in the gut lumen, decreasing its systemic bioavailability (Phillips et al. 2008). Moreover, mycotoxin‐degrading microorganisms present in the binder can biotransform AFB_1_ into less toxic metabolites, limiting its accumulation and reducing oxidative damage in hepatocytes and renal tissues (Zhou et al. 2017). Organic acids may further support hepatic and renal protection by stabilising intestinal microbiota and reducing endotoxemia. Through these synergistic mechanisms, MAG minimised the pathological organ enlargement typically associated with aflatoxicosis.

The spleen, bursa and thymus are important components of the poultry immune system and can indirectly assess the immune status of poultry. While the current results did not show any significant effect of AFB_1_ on the immune organ index (spleen, bursa and thymus), this was confirmed by a previous study (Gómez‐Espinosa et al. 2017). Gómez‐Espinosa et al. (2017) reported that feeding 6‐day‐old turkeys with a diet containing AFB_1_ and AFB2 for 2 weeks did not show any changes in the weight of the spleen and bursa, but a severe decrease in lymphoid cells was observed in the bursa and spleen from the histopathological examination of AF organs. This also means that 1 mg/kg AFB_1_ may have caused organ damage in broilers, but the damage was not severe enough to cause a change in the organ index.

Aflatoxin poisoning in broilers can be detected by biochemical and haematological changes before clinical signs appear (Aravind et al. 2003). Several reports indicate a decrease in total protein and serum albumin in animals exposed to aflatoxin (Osweiler et al. 2010). It has been reported that a decrease in total protein in aflatoxin‐induced liver damage is a sign of toxin‐induced liver damage (Zabiulla et al. 2021). Chen et al. (2014) showed that aflatoxin consumption significantly reduces serum albumin, total protein and globulin levels. In the present study, serum total protein and albumin concentrations in the aflatoxin group were significantly reduced compared to the control group (Table 5). Due to the destructive effect of toxins on liver tissue, the synthesis of plasma proteins, especially albumin, is reduced. The decrease in albumin in the experimental group containing aflatoxin is probably due to liver and kidney damage caused by the introduction of this toxin in the chicken diet. Amongst the factors that cause the decrease in total protein, we can mention the increase in protein metabolism during acute stress, the decrease in protein synthesis due to liver damage and the decrease in protein reabsorption due to kidney damage (Shi et al. 2006). The MAG used alleviated the adverse effects of aflatoxin on serum total protein levels. The decrease in total protein under the influence of aflatoxin and the effects of MAG on serum parameters are consistent with previous studies in this field (Barati et al. 2018). Arab Abousadi et al. (2007) reported that the addition of yeast cell wall to the AFB_1_‐contaminated diet prevents changes in the levels of these indicators.

Increased liver enzyme activity has been reported as a sensitive serological indicator of liver and kidney toxicity (Hatipoglu et al. 2024). Increased levels of AST, ALT and ALP may be a sign of degenerative changes in liver tissue (Aravind et al. 2003). In general, these enzymes are not specific to plasma, but rather exist mostly within cells and enter the plasma as a result of cell damage. One of the reasons for the increased activity of these enzymes could be damage to hepatocytes (Ijaz et al. 2023). In the present experiment, AFB_1_ increased the amount of ALT and AST compared to the control group, which is an indicator of liver damage (Table 7). Adding toxin binder to the aflatoxin‐containing diet reduced serum ALP and AST activities compared to the AFB_1_ group. The addition of adsorbents to the AFB_1_‐containing diet reduced the serum concentrations of these enzymes, indicating the effectiveness of different adsorbents in reducing the effect of AFB_1_ (Raju and Devegowda 2000). The liver is responsible for the production of most circulating proteins. AFB_1_ affects liver function by damaging liver cells. When liver cell permeability increases or liver cells are damaged, transaminases may be released from liver cells into the blood and increase the activity of serum transaminases. Increased serum AST and ALP indicate cellular (hepatocyte) damage, leading to necrosis or changes in cell membrane permeability and muscle damage due to disruption of cell membranes by lipid peroxidation induced by AFB_1_ (Rajput et al. 2017).

The improvement in blood biochemical parameters by MAG supplementation may be attributed to its ability to limit aflatoxin absorption and attenuate hepatic damage. AFB_1_ commonly reduces serum total protein and albumin concentrations due to impaired hepatic protein synthesis, while simultaneously increasing liver enzyme activities (ALT, AST and ALP) as a result of hepatocellular leakage and tissue injury (Tedesco et al. 2004). Yeast cell wall components in MAG likely adsorbed AFB_1_ within the intestinal lumen, reducing systemic toxin exposure and thereby preserving hepatic protein synthesis (Yiannikouris and Jouany 2002). Aluminium silicates and mineral adsorbents provided an additional binding effect, preventing aflatoxin entry into the bloodstream. Moreover, mycotoxin‐degrading microorganisms could biotransform AFB_1_ into less toxic compounds, reducing oxidative stress and hepatocellular necrosis (Zhou et al. 2017). Organic acids may further support liver function by improving gut health and nutrient absorption. Collectively, these mechanisms explain the normalisation of total protein and albumin levels and the reduction of elevated liver enzymes in broilers fed MAG.

A decrease in antibody titres against IBD was observed in birds fed AFB_1_, and these results were consistent with previous findings by Jahanian et al. (2019). The decrease in antibody titres against IBD when fed AFB_1_ was attributed to regression of the bursa of Fabricius, lymphocytolysis and lymphoid depletion. Dietary aflatoxin increases the activity of specific lysosomal enzymes in the liver and muscle, leading to further antibody degradation (Pasha et al. 2007). The toxin binder moderately improved antibody titres against IBD at Day 42, which may indicate a protective effect against aflatoxicosis. As an indicator of humoral immune responses, serum immunoglobulins such as IgG, IgM and IgA are often determined. Consistent with the observed trend of decreased serum protein content in AFB_1_‐exposed birds in this study, a decrease in IgA was also observed, confirming previous reports (Lai et al. 2022). Not only in chickens, AFB_1_‐contaminated diets have been shown to have a significant effect on serum IgG and IgM, IgA concentrations in mice (Long et al. 2016), pigs (Marin et al. 2002), dairy cattle (Xiong et al. 2018) and humans (Turner et al. 2003). While dietary supplementation with MAG increased serum ALB and IgM, IgA, which is in agreement with previous studies showing that toxin binders can improve immune responses in chickens (Liu et al. 2018). AFB_1_ increases the activity of lysosomal enzymes in skeletal muscle and liver. This effect enhances antibody degradation. Aflatoxin inhibits phagocytic cells of the reticuloendothelial system, which are involved in the processing of antigens, as well as cells of the bursa of Fabricius, which are involved in the initiation of the humoral response (Mesgar et al. 2022). It has been suggested that lymphoid organs are vulnerable to mycotoxins due to the activity of lysosomes and hydrolytic enzymes. In addition, reduced protein synthesis, especially IgG and IgA, may be a reason for the immunodeficiency state caused by aflatoxins (Jahanian et al. 2019).

RBCs are composed of protein. The range of RBC count (4.25–3.19) recorded in this work was within the range of 2.90–4.10 ( x 106 mm^3^) recommended by Rafiu et al. (2019). However, the decrease in RBC count observed in Treatment 1 may be an effect of aflatoxin consumption, as it has been reported by Cassel et al. (2012) that the level of protein and vitamins in the feed should be increased because the toxin affects the synthesis of protein, which in turn is the building block of the RBC component of the blood. Monocytes play an important role in the recovery process, so their number increases during the recovery process. The average monocyte count for a bird is 2% (Rafiu et al. 2019). It is therefore suggested that the increase in monocyte counts of more than 2.00% in Treatments 2, 3, 4 and 5, where the contaminated diets contained binders, activated charcoal contained activated charcoal and Treatment 6 (control), probably indicates that the recovery process is needed, although it may be mild as no mortality was recorded in these treatments. However, the lowest monocyte count (1.00%) obtained from Treatment 1 (contaminated diet without binder) indicates that the birds were more stressed by the poison consumed. Monocytes are a type of WBC that is produced in the bone marrow and enters the blood and migrates to tissues (spleen, liver, lung and bone marrow) where they become macrophages. Monocytes are part of the innate immune system and are associated with intestinal inflammatory processes that, in the case of chickens, reduce productivity (Riahi et al. 2021). A significant decrease in monocyte count was also observed with increasing additive levels. Inclusion of this additive in mycotoxin‐contaminated feed resulted in a decrease in monocyte count, which could indicate less intestinal inflammation, suggesting a beneficial effect for the chickens. Subcutaneous ochratoxin A exposure has been described to increase monocyte levels in broiler chickens (Khan et al. 2019).

The restoration of immune competence and haematological balance by MAG can be explained through the combined effects of its active ingredients. AFB_1_ is well known to suppress humoral immunity by reducing antibody production (IgG, IgM and IgA) and vaccine response (IBD and ND titres), largely due to its cytotoxic effects on lymphoid organs and its ability to impair protein synthesis in B lymphocytes (Qureshi et al. 1998; Sur and Celik 2003). The yeast cell wall fraction of MAG, rich in β‐glucans and mannans, may stimulate the innate immune system and enhance antibody production by activating macrophages and modulating lymphocyte activity (Spring et al. 2000). Mineral adsorbents such as aluminium silicates contribute by reducing systemic aflatoxin load, thereby preserving lymphoid tissue integrity. In addition, mycotoxin‐degrading microorganisms can detoxify AFB_1_ into less immunotoxic metabolites, further protecting immune cells (Zhou et al. 2017). Organic acids may also help maintain gut microbial balance, indirectly supporting immune responses. Regarding haematological changes, the normalisation of RBC counts and monocyte percentages observed in the MAG‐supplemented group suggests that reducing oxidative stress and systemic inflammation mitigated AFB_1_‐induced haematopoietic disruption. Taken together, the synergistic action of these bioactive components explains the improved immune function and stabilised haematological parameters in broilers exposed to AFB_1_.

## 5 Conclusion

Overall, broilers fed diets contaminated with 200 µg/kg AFB_1_ showed poor growth performance throughout the period. These adverse effects of aflatoxicosis may be associated with liver dysfunction, immune dysfunction and inflammation of immune organs. However, supplementation of 1 g/kg MAG counteracted the negative effects caused by AFB_1_, reducing liver dysfunction and immunosuppression and improving growth performance. Further studies are needed to explore the protective roles of the mycotoxin binder MAG in AFB_1_‐contaminated diets in broilers.

## Author Contributions

**Payman Mahmoudi Nasr**: investigation, data curation, writing – original draft, writing – review and editing, software, formal analysis, resources. **Kaveh Jafari khorshidi**: supervision, conceptualization, project administration, validation, visualization, methodology, funding acquisition.

## Funding

The authors have nothing to report.

## Ethics Statement

The authors confirm that the ethical policies of the journal, as noted on the journal's author guidelines page, have been adhered to and the appropriate ethical review committee approval has been received. The authors confirm that they have followed Animal Care Committee and Animal Research Ethics Board approval from Islamic Azad University‐Sari Branch, Iran under the protocol number of IR.IAU.SARI.REC 1404.081 throughout the study.

## Conflicts of Interest

The authors declare no conflicts of interest.

## Data Availability

The data that support the findings of this study are available from the corresponding author, Kaveh Jafari khorshidi, upon reasonable request.
